# Supplementary figures and images for: Genetic mapping reveals Nfkbid as a central regulator of humoral immunity to Toxoplasma gondii
Source: PLoS Pathog. 2021 Dec 6;17(12):e1010081. doi: 10.1371/journal.ppat.1010081 (PMC8675933; doi:10.1371/journal.ppat.1010081)

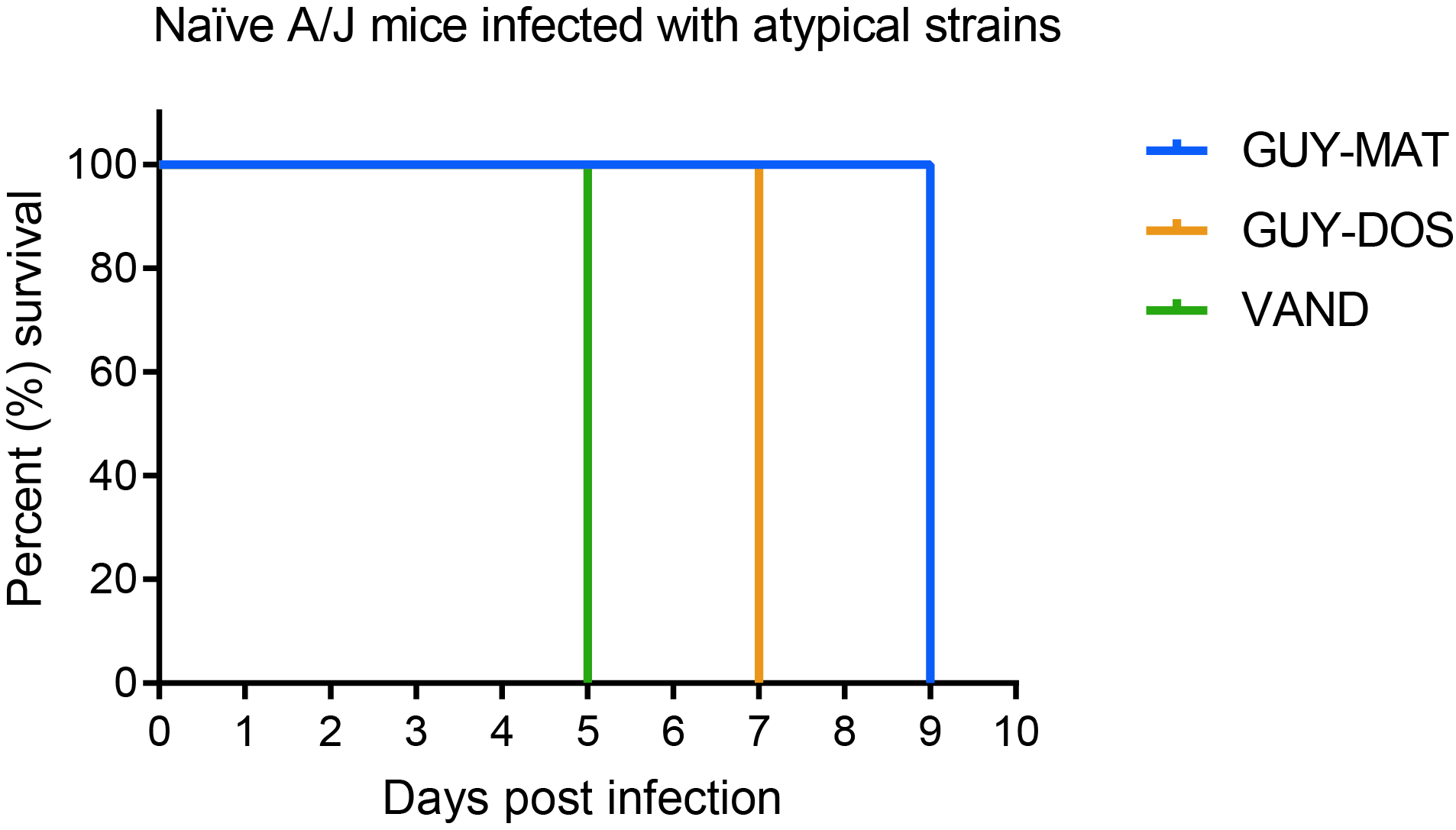

Supplement: S1 Fig — Naïve A/J mice (n = 1) were injected i.p. with the 5x10^4^ tachyzoites of the indicated atypical strains. Survival curves are shown. (TIF) [file ppat.1010081.s001.tif]

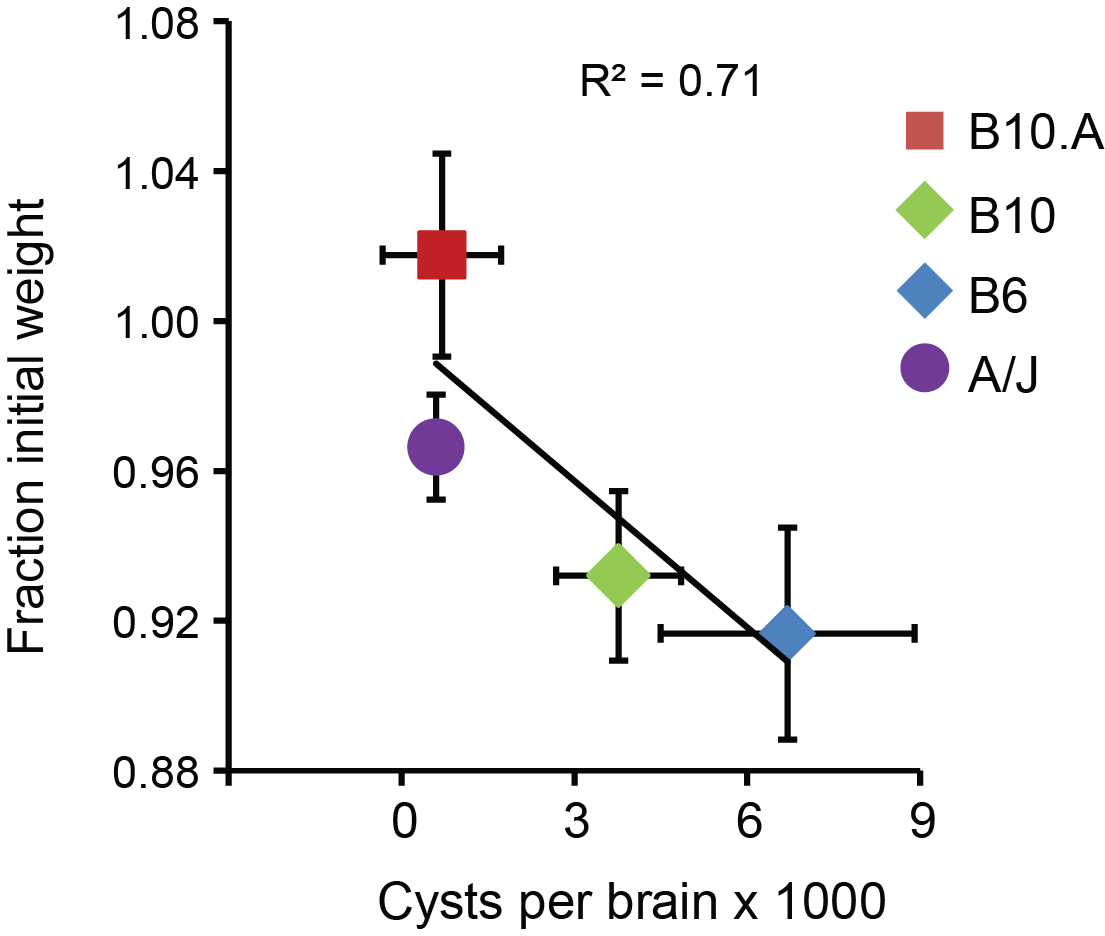

Supplement: S2 Fig — A/J, C57BL/6J (B6), C57BL/10J (B10), and C57BL/10.AJ (B10.A) mice were injected with the type III strain CEP hxgprt- and allowed to progress to chronic infection. Plotted (+/- SEM) is the average cyst number (x 1000) in the brain vs. the average fraction of initial weight, where 1 is the normalized weight on the day of type III injection; the regression value (R2) is indicated. Results are from 3 to 5 mice for cyst numbers (day 42 of chronic infection), and 5–12 mice for weight measurements (day 30 of chronic infection) per mouse strain. (TIF) [file ppat.1010081.s002.tif]

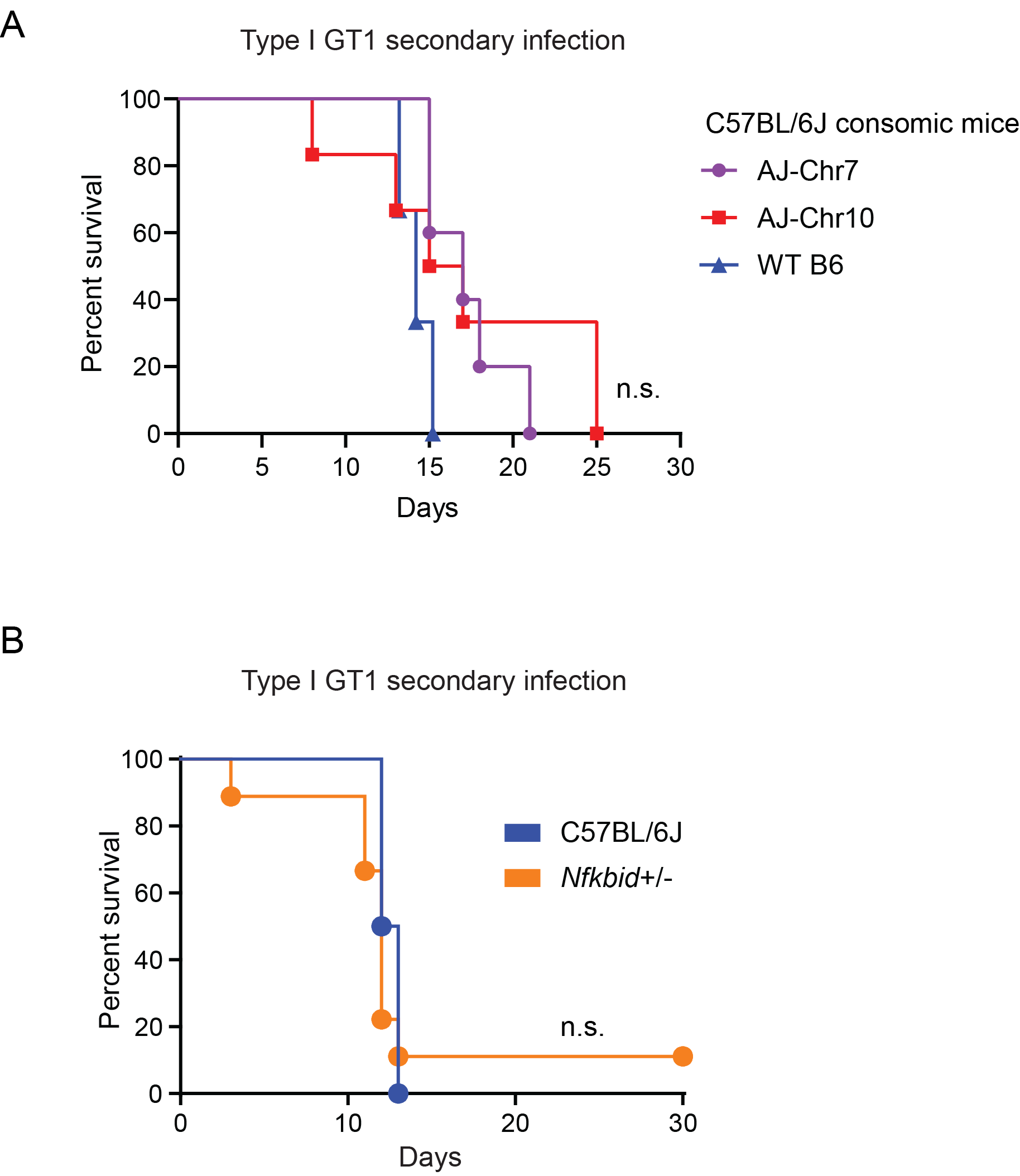

Supplement: S3 Fig — A) Consomic mice of the C57BL/6J background with A/J chromosomal substitutions for chromosome 7 (C57BL/6J-Chr7A/J/NaJ) or chromosome 10 (C57BL/6J-Chr10A/J/NaJ) were infected with the type III CEP hxgprt- strain and allowed to progress to chronic infection. Mice were then given a secondary infection with the type I GT1 strain. B) Survival of Nfkbid+/- (C57BL/6J x bumble F1) and C57BL/6J mice against type I GT1 secondary infection. For A and B, cumulative survival is shown for 2 independent experiments (CSS7 n = 5, CSS10 n = 6) (C57BL/6J n = 4, Nfkbid+/- n = 6); n.s., Mantel-Cox. (TIF) [file ppat.1010081.s003.tif]

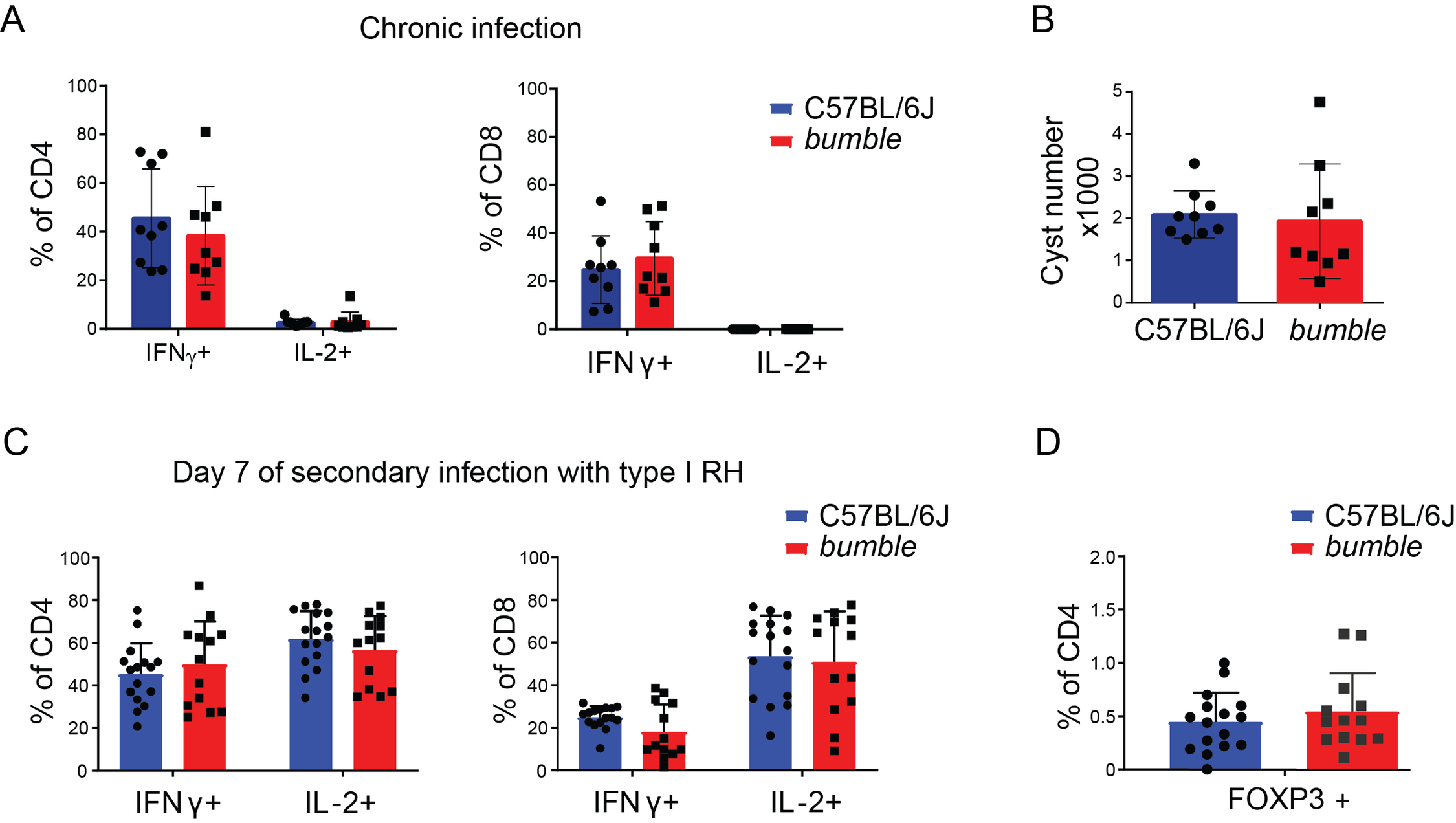

Supplement: S4 Fig — A) Peritoneal CD4 and CD8 T cells from bumble and C57BL/6J mice were assessed between days 32 and 35 of chronic infection with the type III CEP strain by an in vitro recall assay and assayed for intracellular IFNγ and IL-2. In brief, peritoneal cells were harvested and infected with live type I RH parasites for 16 hrs. T cells were assessed for production of IFNγ and IL-2 by intracellular staining and FACS. B) Brain cysts were enumerated between days 32–35 of chronic infection. C) As in A, but T cell recall responses were assessed on day 7 of secondary infection with the type I RH strain. D) Peritoneal T-regulatory cells (CD4+ CD25+ Foxp3+) were quantified on day 7 of secondary infection with type I RH strain. Each dot represents the result from one mouse, and plotted are cumulative averages +SD from 3 experiments for C and D and 2 experiments for A and B; no significant differences were observed between bumble and C57BL/6J mice by unpaired parametric t-tests for data in B-D and the IFNγ response in A; the IL-2 response in A was not significant by a non-parametric Mann Whitney t-test. (TIF) [file ppat.1010081.s004.tif]

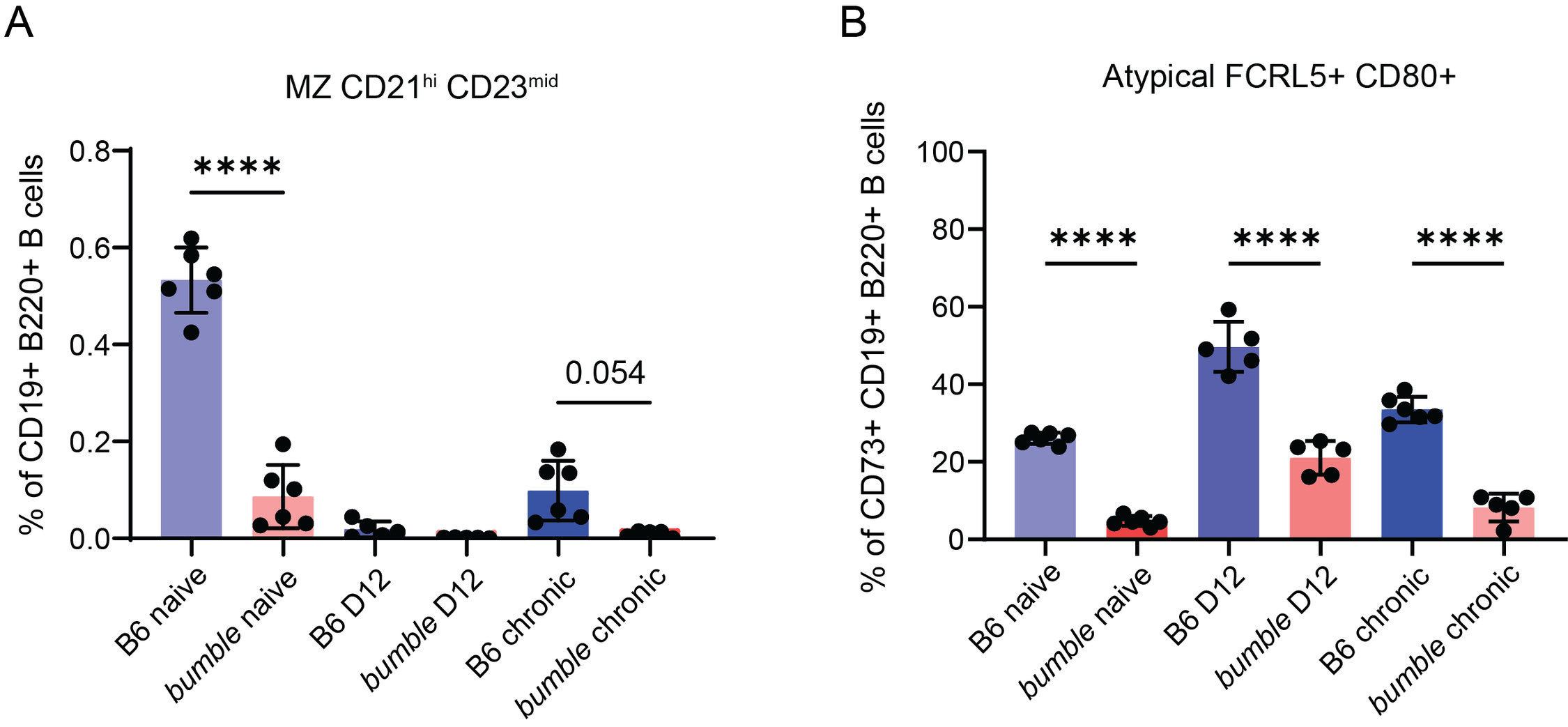

Supplement: S5 Fig — A) Frequency of marginal zone (MZ) B cells (CD21+ CD23mid) among total splenic CD19+ B220+ B cells, and B) frequency of atypical B cells (FCRL5+ CD80+) among total splenic CD19+ B220+ CD23+ CD21mid CD73+ memory B cells, at naïve, d12 of primary infection, and chronic infection with the type III strain in bumble and C57BL/6J mice. Cumulative data from two experiments n = 5–6 mice/condition. Significance was assessed with an unpaired two-tailed t-test; **** P<0.0001. (TIF) [file ppat.1010081.s005.tif]

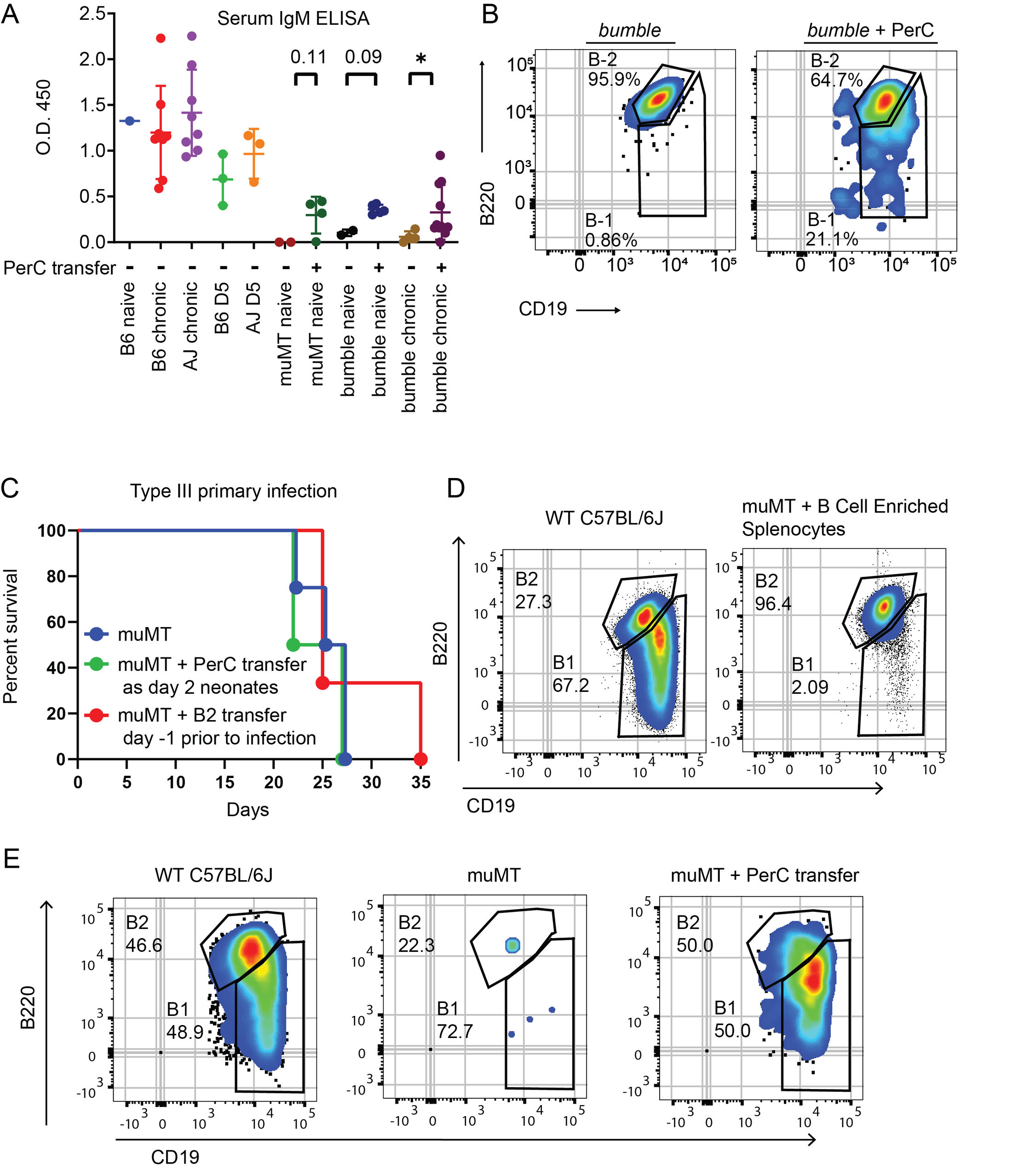

Supplement: S6 Fig — A) Serum IgM from C57BL/6J, muMT, bumble, and A/J mice was measured by ELISA. Serum was harvested from mice either naïve, chronically infected with the type III CEP strain, or on D5 post-secondary infection with the type I GT1 T. gondii strain. PerC transfer (+) refers to mice adoptively transferred 5x10^6^ total C57BL/6J PerC cells as a day 2 neonate. Each dot represents the results from an individual mouse, and plotted is the average +/-SD of the O.D. obtained at 450nm; *P<0.05, unpaired two-tailed t-test. B) Bumble reconstitution of the peritoneal B-1 compartment after neonatal PerC adoptive transfer. Representative FACS plots of peritoneal B-2 cells (B220high CD19+) and B-1 (B220int-neg CD19+) cells from bumble mice with or without PerC adoptive transfer. Shown are mice on day 20 of primary infection with the type III CEP strain. C) B cell deficient muMT mice (n = 3), muMT given WT PerC adoptive transfers as 2-day neonates then allowed to reconstitute for 6–7 weeks into adulthood (n = 2), and muMT given B cell enriched splenocytes (n = 3) 1 day prior to infection with the type III CEP strain were assessed for survival. D) muMT reconstitution of the B-2 cell compartment. WT and muMT with B cell enriched splenocytes (EasySep Mouse Pan-B Cell Isolation Kit, cat# 19844) adoptively transferred 24 hrs earlier, representative FACS plots of peritoneal B-2 cells (B220high CD19+) and B-1 (B220int-neg CD19+) are shown. E) muMT reconstitution of peritoneal B cell compartment after neonatal adoptive transfer. Representative FACS plots of peritoneal B-2 cells (B220high CD19+) and B-1 B cells (B220int-neg CD19+) from WT, and muMT mice or muMT mice with neonatal PerC adoptive transfer. For D and E, uninfected mice are 6–8 weeks of age and numbers indicate the percent of cells that fall within the depicted gate. (TIF) [file ppat.1010081.s006.tif]

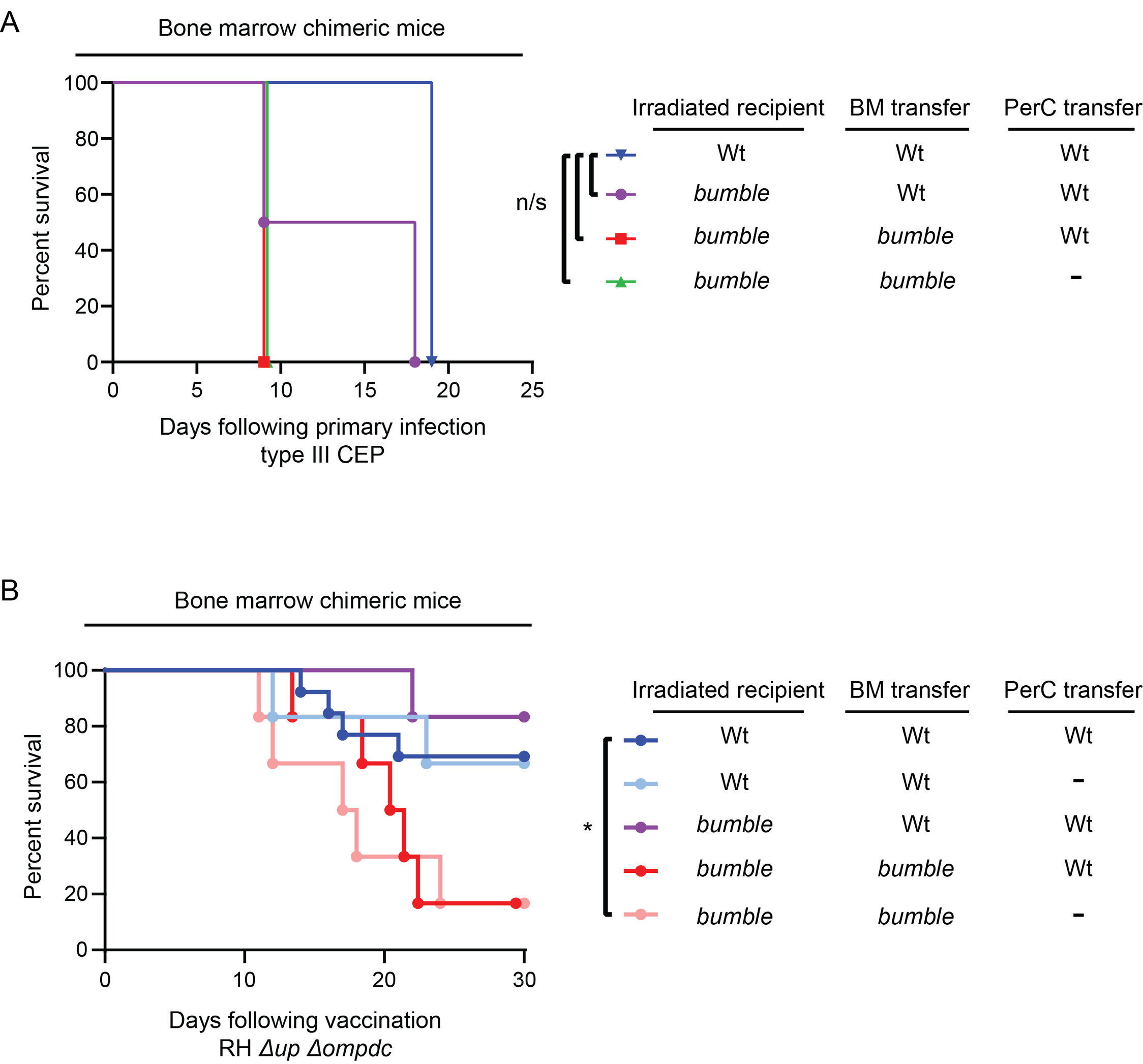

Supplement: S7 Fig — A) Survival of the indicated bone marrow (BM) chimeras infected with the type III CEP T. gondii strain are plotted from a single experiment (n = 2 for bumble recipients per condition; n = 1 for C57BL/6J recipients); n.s., not significant, Mantel-Cox. B) Survival of the indicated BM chimeras vaccinated (106 i.p.) with the uracil auxotroph strain, Rh Δup Δompdc. Results are cumulative from 2–3 separate transfers and vaccinations; (n = 4–9 per condition); * P<0.05, Mantel-Cox. (TIF) [file ppat.1010081.s007.tif]

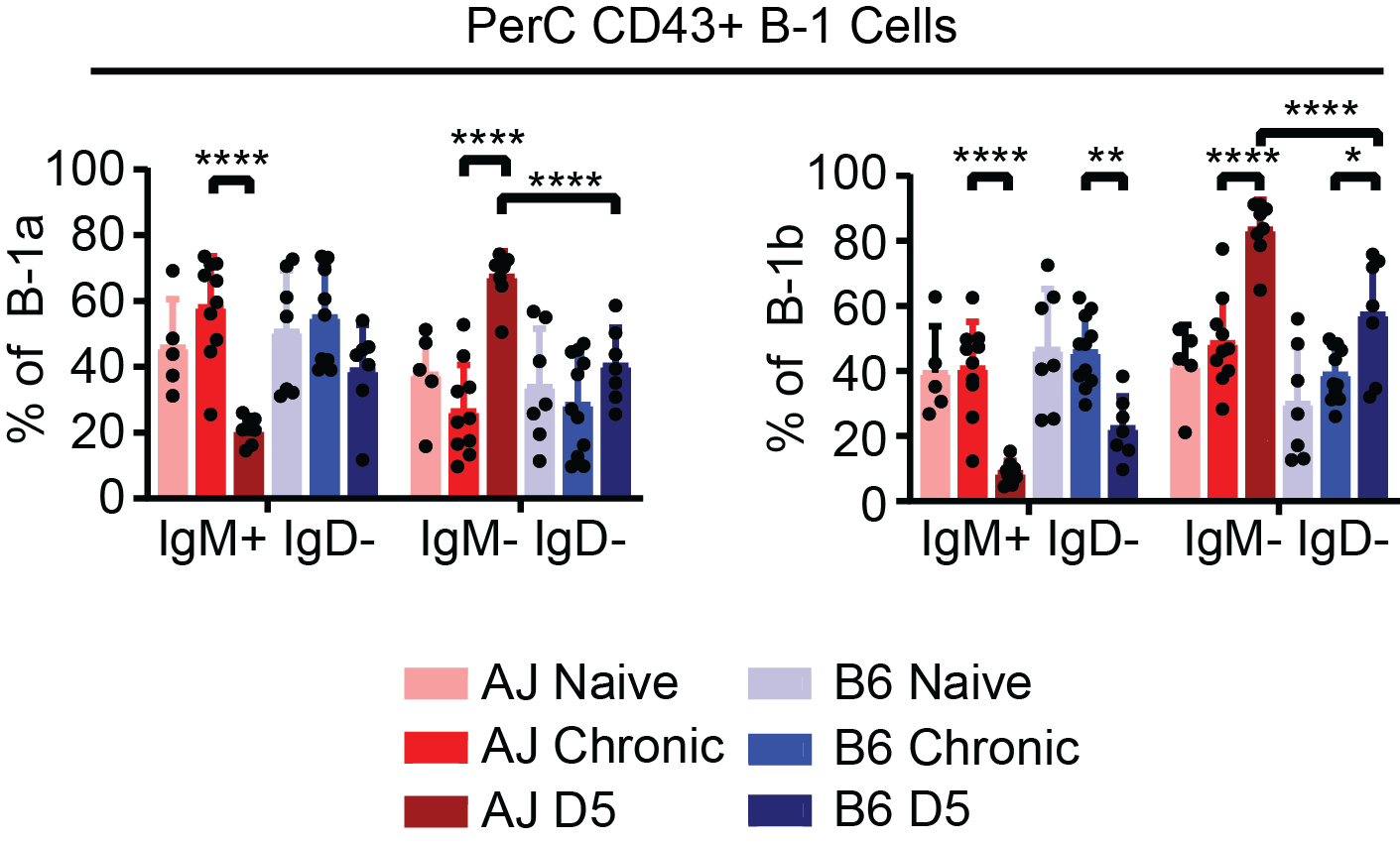

Supplement: S8 Fig — Frequencies of peritoneal (PerC) B-1a (CD5+) or B-1b (CD5-) B-1 cells (CD19+ B220int-neg CD43+) that are IgM+IgD- or IgM-IgD- in A/J and C57BL/6J mice at the indicated infection states. The cumulative average +SD from 2–4 experiments are plotted and each dot represents the result from an individual mouse; P values calculated by 2-way ANOVA with Tukey correction; **** P<0.0001, ** P<0.01, * P<0.05. (TIF) [file ppat.1010081.s008.tif]

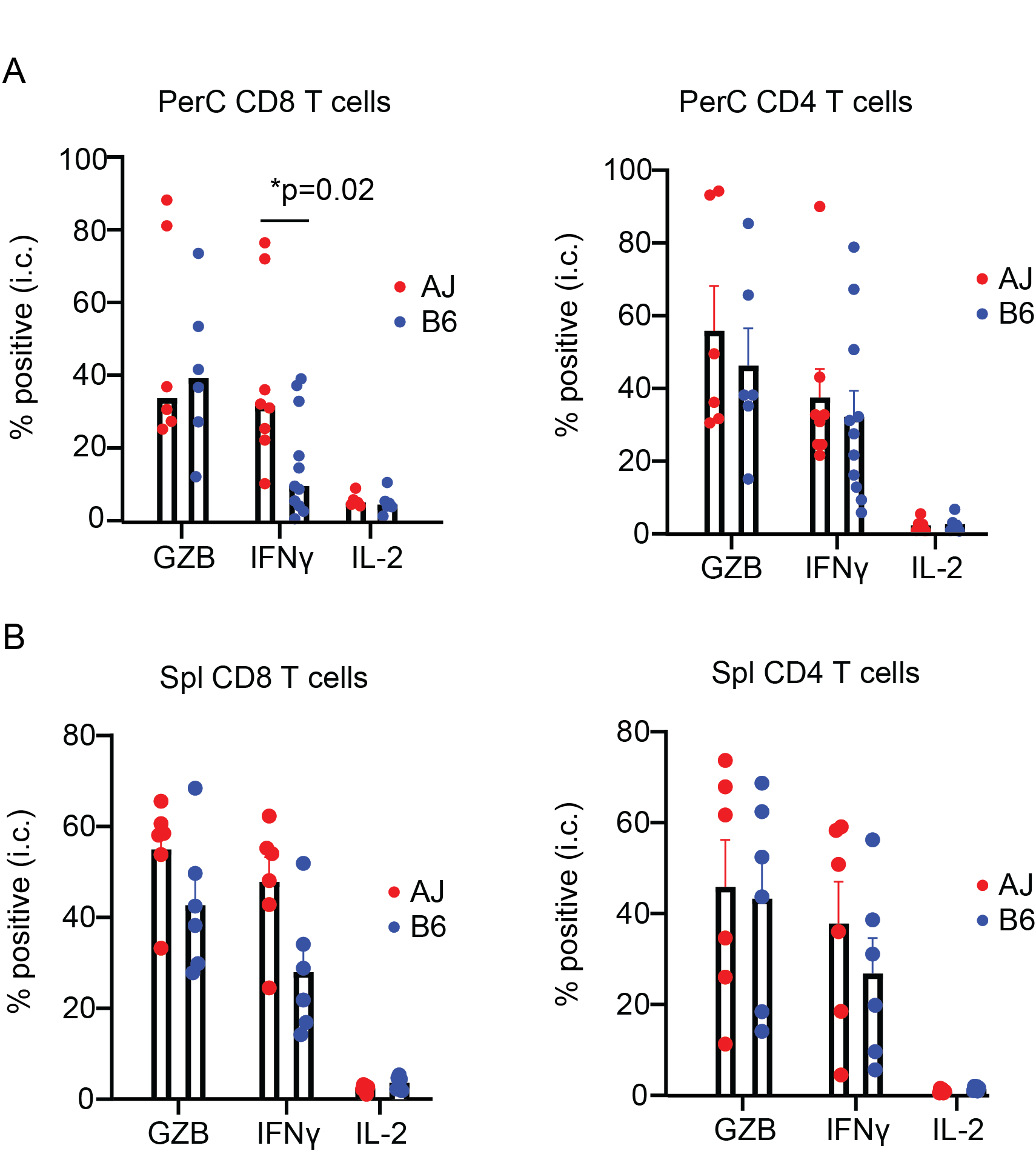

Supplement: S9 Fig — A) Peritoneal, and B) splenic cells were harvested from A/J and C57BL6/J mice chronically infected with the type III CEP T. gondii strain, and infected with live type I parasites for 16 hrs. T cells were assessed for production of granzyme B (GZB), IFNγ, and IL-2 by intracellular staining and FACS. The average frequency +/-SD of positive staining CD4+ or CD8+ T cells (CD3+ CD19-) and cumulative results from 2–3 experiments (AJ n = 8, C57BL6/J n = 11) are shown; * P<0.05, unpaired two-tailed t-test. (TIF) [file ppat.1010081.s009.tif]

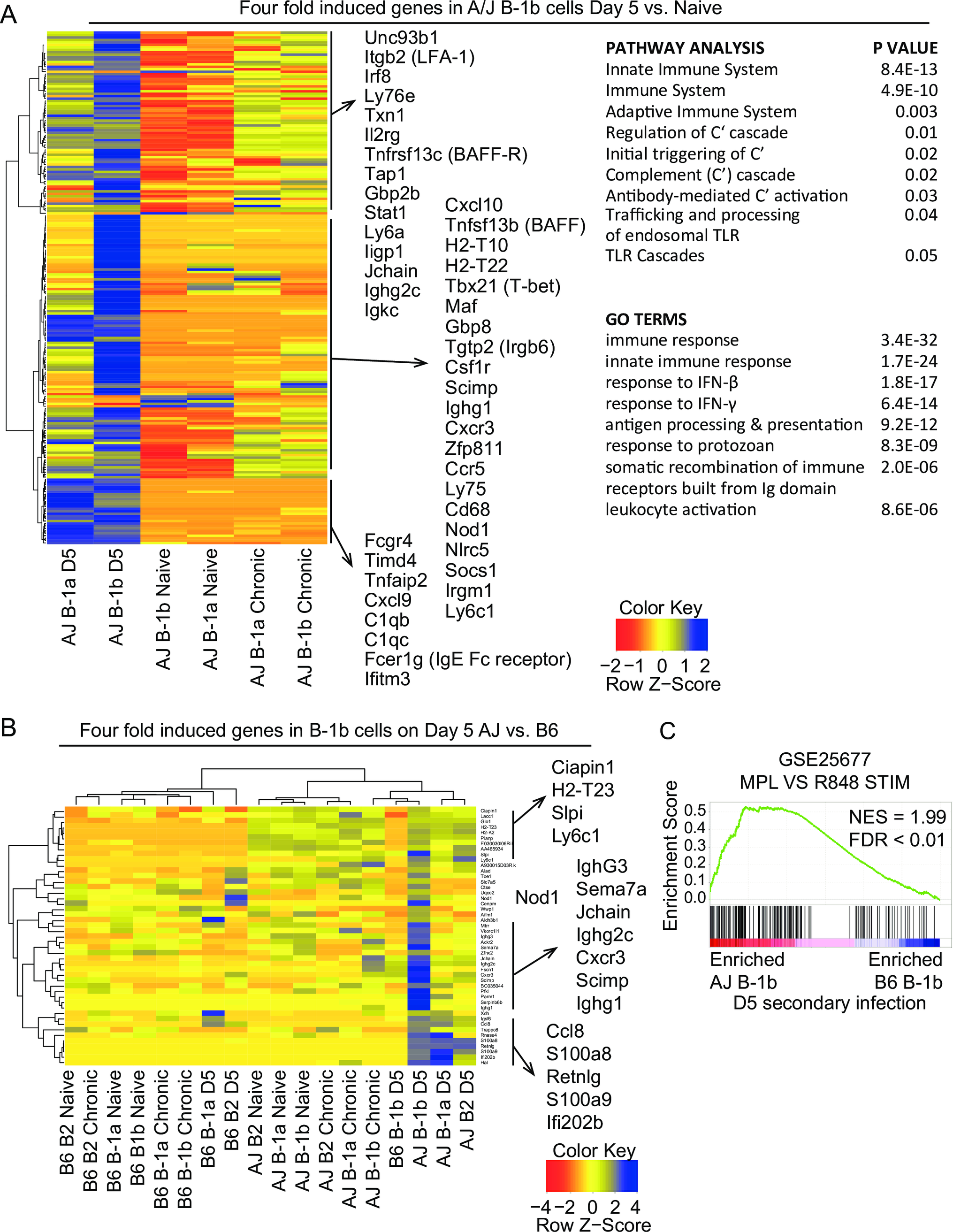

Supplement: S10 Fig — Transcriptomic analysis of peritoneal B-1a (CD19+ B220int-neg CD11b+ CD5+), B-1b (CD19+ B220int-neg CD11b+ CD5-) and B-2 (CD19+ B220hi CD11b- CD5-) B cells from A/J and C57BL/6J mice was performed using 3’-Tag RNA sequencing. A) Genes that were differentially upregulated in B-1b cells on day 5 of secondary infection compared to naïve mice in the A/J genetic background. P values of differentially expressed genes were calculated using the Benjamini-Hochberg adjustment for false discovery rate, and only those genes that survived significance were included in the heatmap. For comparison, all B-1 compartments in A/J mice are shown for this gene set. A/J B-1 Pathway and GO term enrichment was assessed on the genes presented in the heatmap in A. P values for enrichment analysis were adjusted with the Holm-Bonferroni correction. B) A cluster of genes found to be differentially induced in B-1b cells in A/J compared to C57BL/6J mice on day 5 of secondary infection are plotted as a heat map. C) Gene set enrichment analysis of the rank-ordered list of differentially expressed genes between A/J and C57BL/6J B-1b cells at D5 of secondary infection. Gene set depicted was in the top 10 gene sets ranked by false discovery rate (FDR) after investigating MSigDB’s C7: immunologic signatures collection. Enrichment score is the degree of overrepresentation of a gene set at the top or bottom of a ranked list. NES is the enrichment score after normalizing for gene set size. (TIF) [file ppat.1010081.s010.tif]

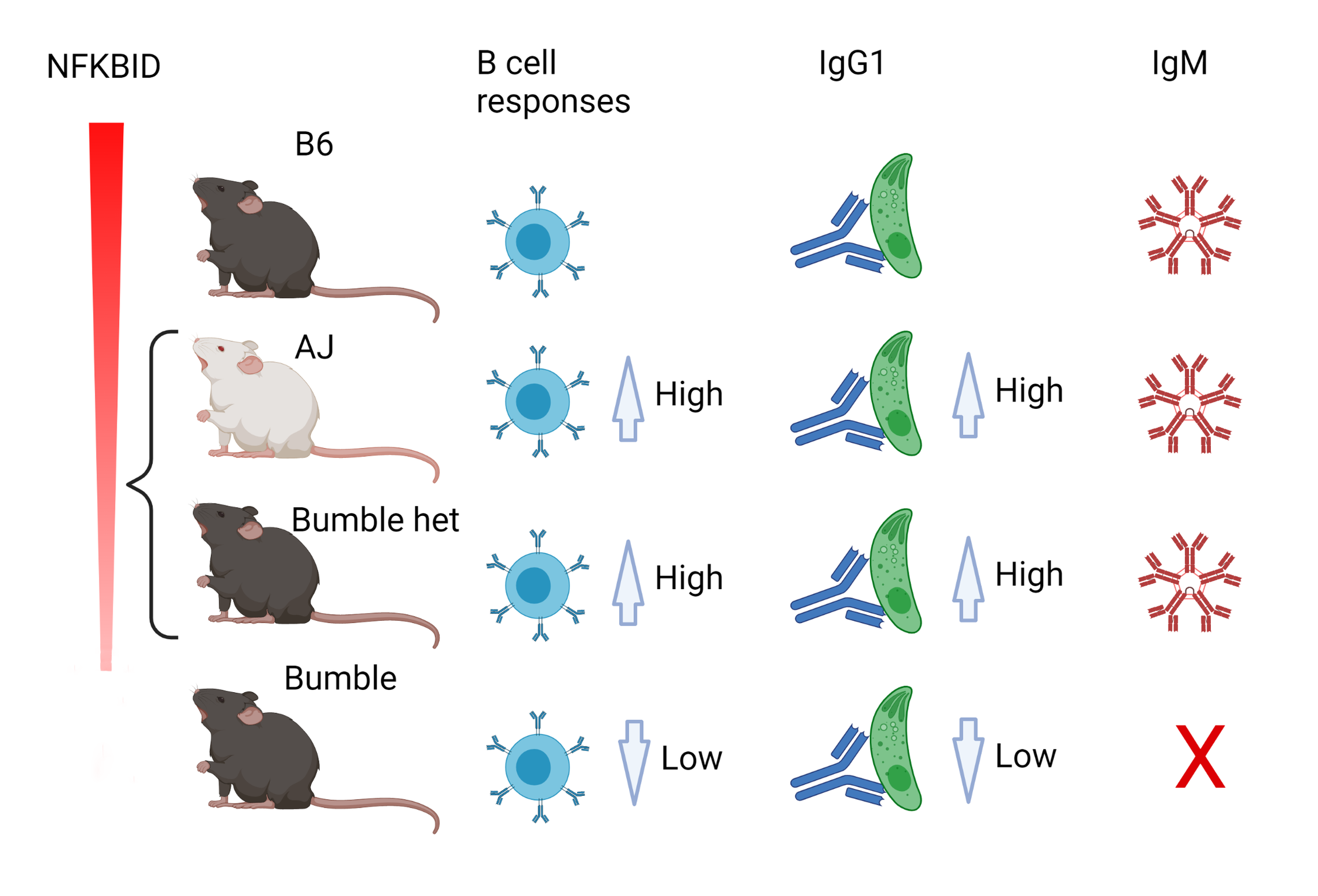

Supplement: S11 Fig — C57BL/6 (B6) mice have high expression of Nfkbid and exhibit B cell responses to T. gondii that produce parasite-specific IgG1 and IgM. In contrast, A/J and ‘Bumble het’ mice (C57BL/6J x bumble F1; Nfkbid+/-) have lower Nfkbid expression and exhibit enhanced IgG1 responses to T. gondii. In the case of ‘Bumble hets’, the enhanced IgG1 response correlates with increased plasma blast differentiation. In the case of A/J mice, overall increased B-1 and B-2 cell responses are observed, though the exact role that Nfkbid plays in B cells in this genetic background is currently undetermined. Finally, ‘Bumble’ mice do not express Nfkbid and have overall poor humoral immunity to T. gondii. Bumble mice produce no parasite-specific IgM and have greatly reduced parasite-specific IgG. There is also a defect in B cell maturation during chronic infection in Bumble mice. We hypothesize that intermediate expression of Nfkbid represents the optimal level for humoral immunity to T. gondii. Schematic created with BioRender.com. (TIF) [file ppat.1010081.s011.tif]
