# Supplementary material for: Genetic mapping reveals Nfkbid as a central regulator of humoral immunity to Toxoplasma gondii
Source: PLoS Pathog. 2021 Dec 6;17(12):e1010081. doi: 10.1371/journal.ppat.1010081 (PMC8675933; doi:10.1371/journal.ppat.1010081)
Supplement: Attachment — Submitted filename: rev pp.docx [file ppat.1010081.s016.docx]

This is an interesting manuscript submitted by Souza et al in which they attempt to demonstrate the role of NFkbid in the generation of antibody response against important intracellular pathogen, Toxoplasma gondii. Initially studies are performed to determine that compared to C57BL/6 mice, A/J strain is highly resistant to secondary parasitic infection. Experiments have been performed to determine to identify NFkbid as an important component required for B cell development. For this purpose, they have used “Bumble mice” which are deficient in NFkbid and demonstrate that the mutant animals are highly susceptible to infection, have a defect in B-2 cell maturation and have down regulated IgM and IgG response. While the experiments performed with C57BL/6 and bumble mice are convincing and demonstrate the importance of NFkbid in the development of B cell response and protection against T. gondii infection are convincing the later studies conducted with A/J mice are correlative. Experiments in which the NFkbid is over-expressed or deleted by Crispr cas9 in A/j mice would have strengthened the manuscript.

Specific comments:

1. Figure 4D. The survival studies with the transfer of PerC to bumble mice although claimed to be significant are not impressive.
2. Figure 2E: Do the vaccinated mice survive beyond day 30.
3. It has been shown that NFkbid is also important for CD8 and cytokine response in A/J mice. These factors need to be ruled.
4. What is the protective role of NFkbid in T cell deficient mice? Can adoptive transfer of B cells from wild type mice protect these animals?
5. Can over-expression of NFkbid on the B cells in C57BL/6 mice optimize the antibody response and protect the animals.
6. Role of NFkbid in the B cell developed during chronic versus acute infection is not clearly delineated.

Minor comment:

Memory CD8+ T cell response has not been only studied with RH strain of parasite. This is not a correct statement and is not supported by references.
