# Supplementary material for: Genetic mapping reveals Nfkbid as a central regulator of humoral immunity to Toxoplasma gondii
Source: PLoS Pathog. 2021 Dec 6;17(12):e1010081. doi: 10.1371/journal.ppat.1010081 (PMC8675933; doi:10.1371/journal.ppat.1010081)
Supplement: Attachment — Submitted filename: Response to Reviewers FINAL.docx [file ppat.1010081.s017.docx]

Dear Prof Jensen,

Thank you very much for submitting your manuscript "Nfkbid is required for immunity and antibody responses to Toxoplasma gondii" for consideration at PLOS Pathogens. As with all papers reviewed by the journal, your manuscript was reviewed by members of the editorial board and by several independent reviewers. In light of the reviews (below this email), we would like to invite the resubmission of a significantly-revised version that takes into account the reviewers' comments.

Reviewer 1 in particular raises several points that the authors will need to address in a revision manuscript.

We cannot make any decision about publication until we have seen the revised manuscript and your response to the reviewers' comments. Your revised manuscript is also likely to be sent to reviewers for further evaluation.

When you are ready to resubmit, please upload the following:

[1] A letter containing a detailed list of your responses to the review comments and a description of the changes you have made in the manuscript. Please note while forming your response, if your article is accepted, you may have the opportunity to make the peer review history publicly available. The record will include editor decision letters (with reviews) and your responses to reviewer comments. If eligible, we will contact you to opt in or out.

[2] Two versions of the revised manuscript: one with either highlights or tracked changes denoting where the text has been changed; the other a clean version (uploaded as the manuscript file).

Important additional instructions are given below your reviewer comments.

Please prepare and submit your revised manuscript within 60 days. If you anticipate any delay, please let us know the expected resubmission date by replying to this email. Please note that revised manuscripts received after the 60-day due date may require evaluation and peer review similar to newly submitted manuscripts.

Thank you again for your submission. We hope that our editorial process has been constructive so far, and we welcome your feedback at any time. Please don't hesitate to contact us if you have any questions or comments.

Sincerely,

Eric Y Denkers
Associate Editor
PLOS Pathogens

Kami Kim
Section Editor
PLOS Pathogens

Kasturi Haldar
Editor-in-Chief
PLOS Pathogens
orcid.org/0000-0001-5065-158X

Michael Malim
Editor-in-Chief
PLOS Pathogens
orcid.org/0000-0002-7699-2064

***********************

Reviewer 1 in particular raises several points that the authors will need to address in a revision manuscript.


Reviewer's Responses to Questions

To the referee panel, here is a summary of the major revisions made to our revised manuscript.

- New data in Supplemental Figure S3 demonstrating similar CD4 and CD8 T cell IFNg responses during chronic infection before challenge in *bumble* and WT mice. Also, similar numbers of cysts are observed between *bumble* and WT mice.
- New data has been added to Figure 5 showing the neutralization and opsonization efficacy of A/J and C57BL/6 sera, where A/J derived sera is superior to B6 in both effector functions.
- We have added more n in Figure 7F demonstrating heterozygous expression of *Nfkbid* promotes enhanced plasma blast (CD138+B220-) differentiation following challenge and the parasite-specific IgM profile is now included in 7C. Also, more n is added to Figure 2F.
- Included the survival data for *Nfkbid+/-* in Figure S2.
- New summary Figure S8 clarifying our model regarding how *Nfkbid* gene expression influences humoral responses to *T. gondii*.
- Made several revisions in the text to clarify requested points and have changed the title of this revised manuscript.
- We solicit our reviewers’ opinion as to whether they think Figures S3 and S8 should be non-supplemental Figures and moved to the main results section.

**Part I - Summary**
Please use this section to discuss strengths/weaknesses of study, novelty/significance, general execution and scholarship.

Reviewer #1: In this manuscript, Souza et al analyze the mechanisms by which mice previously exposed to T. gondii resist a secondary challenge with a more virulent, heterologous, strain. They take advantage of a genetic dissection in mice using susceptible (C57BL/6) vs. resistant (A/J) mice, and they study more specifically the implication in this phenotype of Nfkbid and of different B cell subsets.

This paper addresses an important but largely neglected aspect of anti-T. gondii immunity, namely the contribution of antibody responses in protective immunity upon secondary parasite exposure. It also sheds light on a relatively poorly studied atypical inhibitor of NF-kB (Nfkbid gene encoding for IκBNS), in the context T. gondii infection. Using multiple models of mice and parasite strains, the authors report intriguing and original findings, and the study is clearly the reflection of a tremendous amount of work. However, due to the fact that certain set-ups fluctuate throughout the figures and that some data are somewhat paradoxical (see below), I must say I had a hard time tying all pieces together in a way that made complete sense. The use of a full Nfkbid KO model also comes with obvious limitations.

At this stage, my feeling is that the manuscript has potential but some reformulating/reshuffling, a few additional controls (see below), and a schematics of the working model, would be valuable to clarify and enforce the message.

Please see revisions. We have included a schematic of the working model as a summary supplemental figure, revised the text and added several requested experiments.

Reviewer #2: This study by Souza, Jensen, Baumgarth and their colleagues highlights the complexity of immunological elements that are required for strain-heterologous immunity to parasites, when the priming and challenge strains vary in their levels of virulence and potentially antigen display. Through their thorough analysis of the genetic architecture that controls immune resistance to secondary challenge with virulent strains of T. gondii, they have exposed the underlying layers of complex additive interactions between multiple gene loci and have highlighted the role of Nfkbid, a regulator of NF-kB in regulating humoral immunity to T. gondii. Their rigorous analyses have revealed a cooperative role of B-1 and B2 lineage cells in providing protective humoral immune responses and have dissected their individual and combined roles at steady state and during the dynamics of the infection. Although their analysis of Nfkbid failed to account for the initial observed differences in genetically resistant and susceptible strains of mice, the study still is very significant, as it reveals novel aspects of B cell activation, synergy and dynamics that can only be observed with live parasite infection and challenge scenarios. This study will help spur further detailed and layered analyses of the B cell responses to T. gondii and perhaps other parasitic agents.

We appreciate the positive review. It was new work for us on B cells, which we did not anticipate when we started the initial genetic screen.

Reviewer #3: The strength of the manuscript is that it describes the role of Nfkbid in the development of B cell responses during T. gondii infection which has not been previously reported. The weakness is that the difference between C57BL/6 and A/j mice are not conclusive unless this molecule is over expressed or deleted and the response determined.

We would argue that the weakness of the study is the actual genetic effect of the polymorphism. The impact on phenotypic variance for the chr7 QTL was calculated to be 24%, hence the penetrance of this polymorphism was predicted not to be strong enough to confer survival on its own. Unfortunately, this prediction bore out with chromosomal substitution mice and our attempts to mimic the lower *Nfkbid* gene expression observed in resistant A/J compared to susceptible C57BL/6 mice by generating *Nfkbid+/-* mice. Through our attempts to understand how the A/J *Nfkbid* polymorphism could work, though not enough for survival alone, led us to observe that lower expression of *Nfkbid* in *Nfkbid*+/- mice promoted enhanced plasma blast differentiation and IgG1 responses to *T. gondii*. We reason from our genetic data that survival to secondary infection is controlled by multiple loci, working in an additive manner, and that the IgG1 response is likely one of several other genetic factors in the A/J genetic background. We have made a supplemental schematic figure to help with the conclusions.

**Part II – Major Issues: Key Experiments Required for Acceptance**
Please use this section to detail the key new experiments or modifications of existing experiments that should be absolutely required to validate study conclusions.

Generally, there should be no more than 3 such required experiments or major modifications for a "Major Revision" recommendation. If more than 3 experiments are necessary to validate the study conclusions, then you are encouraged to recommend "Reject".

Reviewer #1: 1. My 1st interrogation is about the contribution of neutralizing antibodies and the respective roles of B-1 vs B-2 cell subsets in resistance to secondary infection. Fig 4A and 4E suggest a beneficial function of B-1 but the RH tachyzoite coating ability of B-1-derived antibodies appears to be much lower than that of B-2-derived antibodies (Fig 4D). How do the authors reconcile these observations?

We made further edits to our discussion. We highlight that low affinity B-1 derived antibodies can have major consequences on ensuing B-2 humoral responses (see referenced review by Blandino & Baumgarth). One mechanism favored but not demonstrated here is that B-2 cells can internalize IgM-antigen complexes via their IgM receptors, which in turn allows MHCII antigen display, T cell help, and full B-2 commitment to humoral immunity. We also recognize that B-1 cells may contribute to the immune response in alternative ways, including cytokine release, NO production and phagocytosis. It is currently unclear how B-1 cells contribute to immunity in the B6 background. In the A/J background, our transcriptomic and intracellular FACS data suggest it is likely through enhanced IgG production. Please see revision to the discussion.

They set-up a nice assay to detect binding by Facs but a caveat is that this assay does not quantify the neutralization capacity of the immunoglobulins. Could they evaluate the neutralization potential of C57BL/6- vs A/J-derived abs, or of B-1 vs B-2-derived abs, in a parasite invasion assay (as in Fig 3B)?

Though we cannot conclusively test the ability of B-1 derived *T. gondii* antibodies to neutralize, we would predict from the data shown in Figure 4 that their ability to block parasite invasion would be much less than B-2 derived antibodies, especially considering that one study suggests IgM as opposed to IgG is the predominant isotype responsible for blocking parasite invasion of cells (see referenced Couper KN et al I&I 2005) and we found the majority of high-affinity IgM antibodies to be B-2 derived (Fig 4D). We have added new requested data to Figure 5, plotting the neutralization capacity and opsonization potential of AJ and B6 derived sera. A/J derived antibodies are superior in both neutralization and opsonization potential compared to B6.

2. The authors argue that a B cell-intrinsic role of Nfkbid regulates resistance to secondary infection. However, in the absence of selective B cell depletion or B cell-specific conditional ablation of Nfkbid, the formal role of Nfkbid within B cells is hard to formally establish.

Thank you. We have edited our discussion to address these concerns. In the first submission, possible B cell intrinsic effects were speculated upon in the discussion. In the revised discussion, we have elaborated upon B cell extrinsic *Nfkbid*-dependent immune responses that could play an important role in our system, not-withstanding Tfh responses. Our data from the bone marrow chimeric mice shows an *Nfkbid* effect in the non-hematopoietic cells that is required for full efficacy of vaccination. Hence, there are B cell extrinsic functions for *Nfkbid* in this model.

One way could be to transfer peritoneal cells from *bumble* mice in the experiment of Fig 4A? If *bumble* PerC cells did not improve survival (contrary to WT), it would provide a more direct proof for the B cell-intrinsic role of Nfkbid.

Whereas we agree a perfect control would be *bumble* into *bumble* at neonatal day 2, in the steady state, B-1 cells are the only self-renewing immune population in the PerC compartment able to reconstitute recipient mice (neonatal day2 transfers). B-2 cells are notoriously bad at long-term reconstitution, and there are few progenitor stem cells in the PerC. Given the lack of B-1a cells in the PerC of *bumble*, it is not clear what residual population (self-renewing stem cell presumably) would be present in the peritoneum. In any case, the *bumble* recipients in 4A would have these same contaminating progenitor cells (if there at all), hence we didn’t do this control. Adult PerC are commonly used for B-1 cell transfer, but apparently (and we didn’t know this prior to this collaborative work with Nicole Baumgarth) there are no perfect B-1 models, in which wildtype mice can be made to completely lack B-1 cells. All other models, including our *bumble* mice, have some sort of genetic deficiency that renders B-1 cells greatly reduced (or absent in the case of *bumble*) but other pleiotropism remains. We are in the process of obtaining *Nfkbid^fl/fl^* mice and intend to generate B-cell specific deletions to directly assess cell-type specific contributions of *Nfkbid*. But such results are a couple of years away.

Mixed BM chimeras with *bumble* BM + B cell KO (e.g. JH-/-) BM could be useful as well to create a system where all B cells are *Nfkbid*-deficient while other cells are in majority WT, but I admit that these experiments are clearly a long shot.

We attempted this exact experiment (unpublished), but unfortunately infected them with CEP before we were aware of the extreme susceptibility of irradiated bone marrow recipients to *T. gondii* infection. Furthermore, susceptibility was observed with the vaccine strain, which was unexpected and shown in Figure S5. We have not returned to bone marrow chimeras for these frustrating reasons, and as mentioned above, we are in the process of obtaining the recently generated *Nfkbid^fl/fl^* mice to make an Mb1-Cre *Nfkbid^fl/f^*^l^ mouse line, which will greatly assist interpretations. That said, irrespective of the B cell intrinsic vs extrinsic effect of *Nfkbid*, we believe we present strong evidence for a requirement for B-1 cells in immunity to *T. gondii* (Fig 4E). Certainly, there are remaining questions elaborated upon in the discussion, but we hope this will suffice for this manuscript.

3. A last important point would be to determine if the challenged mice are ‘equally’ chronically infected at the time of GT1/RH challenge. Survival is similar between WT and *bumble* mice but is there a difference in CEP parasite burden in the CNS? One could also imagine that clearance may occur in some mice infected with such a low virulence strain. In any case, knowing the ‘starting’ parasite load before challenge seems important for the following interpretations.

Yes, this is true. Differences in parasite burden will impact many downstream immune functions of the two mouse strains. Differences in parasite burden may also indirectly reflect T cell or microglia impairment. We have revised Figure S3 to include data on cyst numbers and T cell responses between *bumble* and WT mice during chronic infection before challenge. Cyst numbers and the CD8 and CD4 T cell IFNg responses are the same between *bumble* and B6 during chronic infection before challenge. Of subtle note, the IL-2 T cell response is something we mainly observed after challenge and not during the chronic infection stage (Figure S3, S6).

Reviewer #2: (No Response)

Reviewer #3: The key experiment is to overexposes the molecule in C57BL/6 mice and to delete it in A/J mice and then look at the phenotype.

We have clarified our text. Mainly, the overexpressed situation is the B6 phenotype and presumably detrimental, while intermediate expression, observed by qPCR and within our transcriptomic set, is the A/J phenotype and presumably beneficial. We have included a schematic in the supplemental to help convey this hypothesis. At this point creating an *Nfkbid-/-* A/J mouse line is several years away. We hope the revision will suffice. In any event, a gene deletion is not the same as the polymorphism whose protective mechanism is more challenging to prove. Hence, we tried to mimic the lower expression observed in A/J mice by generating *Nfkbid*+/- mice and provide evidence that the *Nfkbid* polymorphism may work via modulating gene expression, which in turn can regulate plasma blast differentiation and IgG1 responses (Fig 7).

**Part III – Minor Issues: Editorial and Data Presentation Modifications**
Please use this section for editorial suggestions as well as relatively minor modifications of existing data that would enhance clarity.

Reviewer #1: • Some experimental set-ups vary across the figures (e.g. RH vs. GT1 challenge, primary infection vs. vaccination). I can understand there are justifications for this but, at the same time, it makes it difficult to be sure that they are looking at the same phenomenon all throughout the manuscript. The authors have put a lot of efforts trying to define the determinants of resistance to a lethal secondary infection. But they encounter difficulties working with highly virulent strains in laboratory mice that are known to be very susceptible, which forced them to revise some of the set-ups (vaccinate instead of infect irradiated mice). In a sense, one is left to wonder what the contribution of humoral responses, B cell subsets, and Nfkbid, is in a more ‘simple’ setting, for example a challenge with a high dose of the same (CEP) strain. Such a more ‘simple’ approach would have also helped set the stage to better understand the more complex experiments described here. Perhaps these data already exist? If so, could they remind them to the reader?

In the lead-up publication to this study (Jensen et. al. mBio 2015) we tested a variety of challenge situations and found that C57BL/6J mice were highly susceptible to most heterologous and homologous challenges. Perhaps the easiest (and unexpected) result to explain is that mice immunized with the type I RH d-*up* d-*ompdc* vaccine strain cannot be protected against another type I stain, GT1. The type I strains are clonal, and these two strains (GT1 and RH) differ by ~100 SNPs and a few deletions. The type I RH strain is widely studied in molecular parasitology due to its rapid growth rates, very long survival times when extracellular and ease of use for genetic manipulations. It has been “lab adapted” over its 70-year passaging history, but something was lost during this time which rendered the type I RH strain avirulent during secondary infections (vaccinated or chronically infected). However, in a primary infection the RH strain still retains a lethal dose of 1 parasite as other type I strains do. When we genetically mapped for parasite genes that allow the type I GT1 strain to be virulent during secondary infections it turned up the *ROP5* locus, which encodes the primary virulence factor *T. gondii* uses to resist murine IFNg-induced IRG system aimed at parasitophorous vacuole (PV) destruction. Of note, this locus is identical between RH and GT1, so though resisting the IFNg-IRG system is necessary *it is not sufficient* for causing lethal secondary infections. Indeed, this is what we observed in our analyses of multiple different virulent *T. gondii* strains. Mainly, that all parasite strains expressing virulent combinations of ROP5 (and ROP18 another IRG antagonizing parasite virulence factor) were able to cause morbidity and superinfect the brains of the challenged survivors, but death of the mice was not always observed. Hence, immune evasion is a function of ROP5, and sterile immunity is certainly compromised by antagonizing the IFNγ-IRG system, but there are other parasite genes and perhaps environmental factors that contribute to a lethal outcome for vaccinated or chronically infected mice.

This project started because we were unsure whether those other factors might be due to differences in immune recognition of the primary and secondary infection strains during a heterologous challenge. Of note, the CEP strain is avirulent with a lethal dose of >10^5 because it does not express ROP18 due to an insertion in its promoter. This strain can be made virulent by transgenic expression of ROP18 (CEP+ROP18) which in turn allows it to antagonize the IRG system becoming as virulent as type I strains with a LD~10 parasites. When using the CEP+ROP18 transgenic strain to challenge CEP chronically infected C57BL/6 mice, this homotypic challenge did not cause a lethal outcome, but a high frequency of superinfection was observed (this is probably the simpler situation you were asking about). Hence, homotypic challenges are likely much better for disease outcome in immune individuals, but this isn’t always the case. For counter example, the atypical strain MAS, which resists the IRG system as well as type I strains and is equally virulent during primary infections, is able to kill C57BL/6 mice given a chemotherapeutic vaccine with MAS (i.e. inject mice with live MAS at two-week intervals when on anti-parasitic drug). Because this vaccine approach was sufficient to protect mice against RH but not MAS, failure of immune recognition of mis-matched polymorphic antigens can’t fully explain the immunity defects in C57BL/6 mice (all of this and more is in the Jensen et al mBio paper if interested). We added a sentence in the introduction to elaborate these points.

We chose to perform genetic mapping to identify a genetic determinant strong enough to confer survival against highly virulent challenges, and as such used this particular strain combination of primary (CEP) and secondary infections (GT1) for this study. Had we chosen the strain CEP+ROP18 for the screen, all RI would have survived, and we would have had to map traits like weight loss or superinfection which has greater variability and would have lowered the LOD scores and widened the confidence intervals. Had we chosen a highly virulent atypical strain like MAS, we would have mapped the H-2 locus because it is controlled by A/J H-2 genes (Fig 1A, 1C). This is what we didn’t want, and why we screened all B10.A mice prior to the genetic mapping experiment.

One can reason that immunity generated by natural infection is superior and more long lasting than that elicited by vaccination. The best way to do this in mice is giving type III CEP infections. And certainly, the normal occurrence for animals and humans living in the highly endemic regions of South America would be that of continual exposure to heterologous strains, where *T. gondii* is extremely diverse and lacks the clonal population structure observed in Asia, North America, and Europe. Hence, this model mimics such situations, and we agree, is not simple. However, in a simpler situation shown in Figure 3, we observe the impact of *Nfkbid* deficiency during primary CEP infection which results in drastically reduced B-2 cell maturation and antibody recognition of *T. gondii*. All this prior to the heterologous challenge.

Long winded, but I hope this clarifies.

• Unless I misunderstood, I see a disconnect between the genetic dissection pointing to Nfkbid, among several other genes, as a polymorphic gene potentially involved in A/J resistance, and the findings obtained with Nfkbid KO mice in a C57BL/6 background. On the one hand, Nfkbid is required for resistance in C57BL/6 but on the other hand, the naturally resistant A/J display higher levels of Nfkbid. Is it not a counterintuitive observation? I think such puzzling data would deserve to be discussed more thoroughly. If they have a reconciling hypothesis, it would also greatly help to illustrate it on a working model schematics.

Yes, conveying our observations in a clear schematic was needed, and we have added one in the supplemental. To clarify, C57BL/6J mice have higher expression of *Nfkbid* compared to A/J and therefore the prediction is that overexpression is detrimental. Hence, we generated *Nfkbid* +/- mice to best explore how intermediate expression, perhaps analogous to A/J mice, might confer better immunity. We have revised our description of the data and have included a summary figure to convey our hypothesis that intermediate expression of *Nfkbid* would be predicted to be beneficial, possibly through enhanced plasma blast differentiation and IgG1 responses. To use a silly analogy, *Nfkbid* expression and immunity have a goldilocks relationship, the porridge must not be too hot nor too cold but warmly in the middle.

• In my opinion, the title oversimplifies the data presented here. It does not convey the seemingly complex role of Nfkbid (more a regulator than a positively required element) and the fact that most experiments addressed the resistance to the acute phase of a secondary infection, rather than the general immune responses to T. gondii, or the control of parasite latency.

Thank you for pointing this out, we agree. We have revised the title. We hope it conveys a larger picture of the results and approach presented.

“Genetic mapping reveals *Nfkbid* as a central regulator of humoral immunity to *Toxoplasma gondii”*

• Beside generating a humoral response, CEP primary infection likely induces memory T lymphocytes, which could also be involved in the resistance that is observed against secondary type I challenge. The intracellular cytokine stainings presented in Fig S3 are informative but in my opinion, they may be biased by strong differences in acute phase reported in the two models (Fig 2E), which likely lead to distinct levels of inflammatory cytokines and lymphocyte activation. To more formally exclude a role for T cells in the phenotype, I think it would be important to verify that the T cell responses elicited in the two groups are similar before challenge. Perhaps this could be done by restimulating peritoneal or spleen cells with CEP-infected DC, and/or with endogenous T cell antigens from T. gondii.

We agree. We have revised Figure S3 and analyzed both the T cell recall response prior to challenge and the parasite burden between *bumble* and WT mice during chronic infection. Similar IFNg CD4 and CD8 T cell responses were observed, and similar parasite cyst numbers were detected in both mouse strains.

• I may have missed it but the justification for focusing on Nfkib after the genetic dissection is not obvious.

There were several clues that *Nfkbid* was the likely gene candidate in the Chr7 QTL region. As described in the text, it was one of the most polymorphic genes between A/J and B6 in the confidence region of the chr7 QTL, and it sat in between the genetic markers that flanked an imputed maker with the largest LOD score in this region. (*The imputed marker is a statistical prediction testing hypothetical markers between two real ones and calculating what the LOD would be. In our case, it generated a higher LOD score, because there would have been AJ/B6 chromosomal crossovers between the two real genetic markers with the right alignment of alleles and survival phenotypes somewhere). Importantly, it was also one of the few genes in the chr7 QTL that had known immunological function. When we were screening the RI lines, we began noticing enhanced B-1 responses in the A/J background and hence pursued this gene candidate. Our rubric is not exact, and certainly there are other QTLs generated by our screen that are less clear in revealing the causal gene variants. We noted *Tspan8* in the chr10 QTL, but how it relates to immunity is speculative and it is not expressed in B cells (unpublished) or most other lymphocyte populations (immgen.org). This epithelial expressed gene likely promotes lymphocyte recruitment that might be important for memory development or for rapid deployment of lymphocytes upon challenge, but we don’t know. There is an anti-sense long non-coding RNA to *Tspan8* that shares many of the mutations, so there is certainly much to learn here.

What do the non-synonymous SNP exactly change to the protein? Do they introduce premature stop codons?

In terms of NFKBID polymorphisms, there are 2 NS-SNPs between A/J and B6, but none are stop-codons. The substitutions (130 H->Y, 163 R->L) are predicted to sit between ankyrin repeat domains on loops bridging the domains. Instead, there are multiple intronic and importantly, 3’ UTR SNPs between A/J and B6 *Nfkbid*, which led us to test whether differences in gene expression between mouse strains might account for how the polymorphism works functionally.

What do they do with other candidate genes? Is their implication ruled out, how?

The QTLs bore no evidence for epistasis using ‘2-D scans’ where all possible combinations of 2 markers are assessed for survival correlation, or interactive QTLs where the QTL is tested for influence by all other markers. As mentioned in the text, an additive-QTL model including all four QTLs best fit the data compared to any lesser combination of them (P<0.02, ANOVA). In other words, a summation of each of the mutations is predicted to eventually build the survival phenotype.

• Fig S1: inconsistency of symbol between legend and graph for B6.

Symbols are changed.

• Lines 173, 308 & 335: misformatted ref

Refs are fixed.

• Fig S1. What does the regression value represent? Why not applying a statistical test to compare the samples?

I’m pasting below the separated data.

Admittedly, we only looked at cyst counts in one experiment. B6 is different from B10.A in weight, and different from A/J in cyst number after CEP chronic infection (ANOVA multiple comparisons). We felt if we devoted 10 more mice of each background to these data, we could probably see a clear trend for each variable, but the cost was prohibitive at the time. The main point here is that chronically infected B10.A were overall very healthy at the time of challenge, and yet they succumbed to the infection as a function of the strain type. It wasn’t simply due to issues surrounding T cell exhaustion and loss of control during the chronic infection leading to B10.A susceptibility during challenge. We decided to simply show the trend, that when plotting the two variables, H-2a mice are generally performing better than the H-2b mice with a CEP infection.

• Fig 1D. In my understanding, applying a Fisher’s test on percentages is not appropriate. Rather than showing the fraction of mice superinfected or not, the authors could represent the actual number of mice in each category and then apply the Fisher’s exact test.

Thank you. You are correct, this was an oversight on our end. We re-plotted the actual numbers and the Fisher’s exact test performed.

• Fig 2F: was the normality hypothesis verified to be able to use a parametric t-test?

The *bumble* values passed all normality tests but the B6 values only passed D’Agostino Pearson test, as many values were zero. Also, we have updated the plot in 2F. The original had only 11 *bumble* mice but now report 13, and B6 has 16 values. The updated stats are **** p=<0.0001 (unpaired t test parametric; non-parametric returns the same value).

• Fig S3. I do not understand why the authors made a correction for multiple comparisons. Is the purpose not to compare WT and *bumble* per cytokine? Same as above: was the normality hypothesis verified to use a parametric t-test?

Thank you. This was a mistake of our data entry within Prism. There was no need for multiple comparisons between two samples. All data in Figure S3 B, C, D passed normality tests, none were significantly different (unpaired non-parametric t test). In Figure S3A, the IFNg data passes normality tests, while the IL-2 did not pass, given that there are many zeros. Instead, the IL-2 responses are not significant by unpaired non-parametric Mann Whitney t-test.

Reviewer #2: (No Response)

Reviewer #3: None

Other reviewer comments (reviewer #3 from correspondence with PLOS editors but not sure if this is a reviewer #4)

This is an interesting manuscript submitted by Souza et al in which they attempt to demonstrate the role of NFkbid in the generation of antibody response against important intracellular pathogen, Toxoplasma gondii. Initially studies are performed to determine that compared to C57BL/6 mice, A/J strain is highly resistant to secondary parasitic infection. Experiments have been performed to determine to identify NFkbid as an important component required for B cell development. For this purpose, they have used “*Bumble* mice” which are deficient in NFkbid and demonstrate that the mutant animals are highly susceptible to infection, have a defect in B-2 cell maturation and have down regulated IgM and IgG response. While the experiments performed with C57BL/6 and *bumble* mice are convincing and demonstrate the importance of NFkbid in the development of B cell response and protection against T. gondii infection are convincing the later studies conducted with A/J mice are correlative. Experiments in which the NFkbid is over-expressed or deleted by Crispr cas9 in A/j mice would have strengthened the manuscript.

Specific comments:

1. Figure 4D. The survival studies with the transfer of PerC to *bumble* mice although claimed to be significant are not impressive.

As performed the data is statistically significant in 4D (4 - 9 mice per chimera), though n for the *bumble* -> *bumble* transfers is low (n=4) due to the unexpected finding that the uracil auxotroph strain can be lethal in irradiated bone marrow chimera mice. Hence, B-1 cells are playing an important role in immunity, just not without assistance from other compartments.

Might the question refer to the experiment in 4A? Where PerC transfers into neonate *bumble* mice did not elicit robust protection, simply extending time to death (significant by Mantel-Cox). We reason this occurred because no *Nfkbid*-sufficient B-2 responses and other *Nfkbid*-dependent mechanisms were present in these recipients.

2. Figure 2E: Do the vaccinated mice survive beyond day 30.

To clarify in Figure 2E, these mice are chronically infected with CEP (not vaccinated with the replication deficient strain). Yes, both *bumble* and C57BL/6J survive beyond day 30. We don’t see the progressive wasting of C57BL/6J or *bumble* mice infected with CEP as observed with mice infected with certain type II ME49 strains. The longest we have observed CEP primary infection in our hands has been day 56 and the mice are fine at this stage. In terms of typical kinetics with CEP infection, I’m pasting this graph of weight loss to show that mice after the acute phase of the infection (days 5-16) will rebound and recover in terms of overall health.

We do lose ~5% of CEP infected B6 and *bumble* mice during the early phase of infection (days 8-15) as shown in Figure 2C, but beyond this time of CEP infection the mice look equally well.

3. It has been shown that NFkbid is also important for CD8 and cytokine response in A/J mice. These factors need to be ruled.

In the original supplemental Figure S6, we analyzed the CD8 and CD4 T cell responses in the A/J and B6 backgrounds. A general trend of increased CD8 T cell IFNg production was noted in the PerC of A/J mice, but the CD4 T cell IFNg responses were comparable in the spleen and PerC of A/J and B6 mice. We do agree that we have not studied the role of *Nfkbid*-dependent T cell responses in A/J mice as we did not backcross the *bumble* mutation into A/J animals. And it is possible that there are some QTLs whose polymorphic gene(s) confer enhanced CD8 T cell memory responses to *T. gondii*. We have not specifically done genetic mapping for T cell effector function, which could be revealing. In fact, the reason we setup this secondary survival screen was to find new polymorphic regulators of T cell responses. However, at least for the Chr7 QTL, it is likely working via the regulation of humoral responses. That the IFNg/IL-2 T cell response to *T. gondii* is identical in *bumble* vs WT mice (Figure S3), suggest at least these phenotypes are not underlying *Nfkbid*-dependent immunity.

Finally, as mentioned to the other reviewers, we have modified our discussion to indicate that there are certainly other B cell extrinsic functions for *Nfkbid*. We mainly discussed B-cell intrinsic functions for *Nfkbid* in the original submission, but indeed, the bone marrow chimeric mice indicate a role for *Nfkbid* in non-hematapoetic compartments indicating that *Nfkbid* is important in multiple compartments. Please see the revisions to the discussion.

4. What is the protective role of NFkbid in T cell deficient mice? Can adoptive transfer of B cells from wild type mice protect these animals?

We have not done this experiment but strongly believe B cells would not protect against virulent strains in absence of T cells. The experiments originally performed by Larry Johnson’s group suggest that vaccinated but not un-vaccinated muMT mice survived RH challenge only when they received immune serum (Sayles et al. I&I 2000). In other words, serum alone is insufficient for protection but must be layered into existing memory CD8 T cell responses.

In our model of inducing immunological memory with CEP primary infection, depletion of CD8 T cells renders mice susceptible to RH challenge (Splitt 2019 I&I), which is consistent with the large body of work on the importance of CD8 T cell mediated memory being a requirement for protection in vaccinated or type II infected mice. Our main point is that in addition to the CD8 T cell memory response, to survive virulent challenge the response must be layered with enhanced humoral immunity.

5. Can over-expression of *NFkbid* on the B cells in C57BL/6 mice optimize the antibody response and protect the animals.

While it is possible that over-expression of *Nfkbid* could be beneficial to humoral responses, our data suggests that optimized antibody responses are correlated with intermediate expression of *Nfkbid,* as the resistant A/J mouse has lower levels of *Nfkbid* transcript relative to B6. Overexpression comes at the cost of IgG1 class switching and plasma blast differentiation, as *Nfkbid*+/- mice have improved responses relative to B6 (Fig 7). We have included a supplemental schematic summarizing this data and clarified the text surrounding this data.

6. Role of NFkbid in the B cell developed during chronic versus acute infection is not clearly delineated.

We previously gave the response kinetics of the B-2 populations in the Figure 3C during early and late time points. The most prominent defects are those occurring with the transitional stage of B cell maturation, and then class switching of the follicular CD73+ B cells. The B-2 maturation defect manifests within the spleen during chronic, but not acute infection in *Nfkbid*-deficient mice. While we did not investigate the development arrest, the phenotype observed is consistent with many observations of *Nfkbid* deficiency reducing maturation rates in transitional B-1 cells (Pedersen 2014) and a T1->T2 transitional defect in B-2 cells resulting in a marginal zone deficiency (Adori 2018). We suspect that chronic *T. gondii* infection requires high B cell maturation output and *Nfkbid*-deficient mice are unable to meet that demand as noted in the original discussion.

We agree more questions could be addressed, including a screen for primary infection susceptibility to intermediate virulent strain type II strains and the corresponding *Nfkbid*-dependent humoral response during this time frame, but perhaps the reviewer can agree that a separate study can address this.

- Memory CD8+ T cell response has not been only studied with RH strain of parasite. This is not a correct statement and is not supported by references.

The statement has been removed.

PLOS authors have the option to publish the peer review history of their article ([what does this mean?](https://journals.plos.org/plospathogens/s/editorial-and-peer-review-process#loc-peer-review-history)). If published, this will include your full peer review and any attached files.


If you choose “no”, your identity will remain anonymous but your review may still be made public.


**Do you want your identity to be public for this peer review?** For information about this choice, including consent withdrawal, please see our [Privacy Policy](https://www.plos.org/privacy-policy).

Reviewer #1: No

Reviewer #2: No

Reviewer #3: **Yes:** Imtiaz A Khan

Figure Files:

While revising your submission, please upload your figure files to the Preflight Analysis and Conversion Engine (PACE) digital diagnostic tool, [https://pacev2.apexcovantage.com](https://pacev2.apexcovantage.com/). PACE helps ensure that figures meet PLOS requirements. To use PACE, you must first register as a user. Then, login and navigate to the UPLOAD tab, where you will find detailed instructions on how to use the tool. If you encounter any issues or have any questions when using PACE, please email us at [figures@plos.org](mailto:figures@plos.org).

Data Requirements:

Please note that, as a condition of publication, PLOS' data policy requires that you make available all data used to draw the conclusions outlined in your manuscript. Data must be deposited in an appropriate repository, included within the body of the manuscript, or uploaded as supporting information. This includes all numerical values that were used to generate graphs, histograms etc.. For an example see here on PLOS Biology: <http://www.plosbiology.org/article/info%3Adoi%2F10.1371%2Fjournal.pbio.1001908#s5>.

Reproducibility:

To enhance the reproducibility of your results, we recommend that you deposit your laboratory protocols in protocols.io, where a protocol can be assigned its own identifier (DOI) such that it can be cited independently in the future. Additionally, PLOS ONE offers an option to publish peer-reviewed clinical study protocols. Read more information on sharing protocols at <https://plos.org/protocols?utm_medium=editorial-email&utm_source=authorletters&utm_campaign=protocols>

*In compliance with data protection regulations, you may request that we remove your personal registration details at any time.* [*(Remove my information/details)*](https://www.editorialmanager.com/ppathogens/login.asp?a=r)*. Please contact the publication office if you have any questions.*
